# Establishment of Singleplex and Duplex TaqMan RT-qPCR Detection Systems for Strawberry Mottle Virus (SMoV) and Strawberry Vein Banding Virus (SVBV)

**DOI:** 10.3390/plants14152330

**Published:** 2025-07-27

**Authors:** Tengfei Xu, Dehang Gao, Mengmeng Wu, Hongqing Wang, Chengyong He

**Affiliations:** 1 Institute of Horticulture, Sichuan Academy of Agricultural Sciences, Chengdu 610000, China; 15833326354@163.com; 2 Key Laboratory of Horticultural Crops Biology and Germplasm Enhancement in Southwest, Ministry of Agriculture and Rural Affairs, Chengdu 610000, China; 3 Department of Fruit Science, College of Horticulture, China Agricultural University, Beijing 100107, China; 13126899720@163.com (D.G.);

**Keywords:** SMoV, SVBV, detection system, TaqMan RT-qPCR

## Abstract

SMoV and SVBV are two major viruses that pose significant threats to the global strawberry industry. Both are latent viruses, making early detection difficult due to their uneven distribution and low concentration in host tissues. Traditional RT-PCR techniques are insufficient for precise and quantitative detection. In this study, TaqMan RT-qPCR detection systems for SMoV and SVBV were established for application in practical production settings, enabling accurate, rapid, and efficient detection of strawberry viruses. When viral accumulation in plants is low, the highly sensitive TaqMan RT-qPCR technique allows for accurate quantification, facilitating the early identification of infected plants and preventing large-scale outbreaks in cultivation areas. The development of a duplex TaqMan RT-qPCR assay enables simultaneous quantification of SMoV and SVBV in a single reaction, improving detection efficiency and providing technical support for risk assessment and effective control of strawberry viral diseases.

## 1. Introduction

Strawberry (*Fragaria × ananassa* Duch.) is a perennial herbaceous plant belonging to the genus *Fragaria* in the family Rosaceae [1]. Strawberry is an important economically cultivated crop [2]. Its fruits are tender, juicy, and have a sweet and tangy flavor [3]. In addition, strawberries are rich in various essential nutrients, such as dietary fiber and trace elements [4,5]. Studies have shown that moderate consumption of strawberries offers multiple health benefits. Viral strawberry diseases are a major factor limiting strawberry yield [6]. These diseases are characterized by their insidious onset, difficulty in treatment and elimination, high transmissibility, and severe impact [7]. Among them, four are considered the most detrimental: strawberry mottle virus (SMoV), strawberry vein banding virus (SVBV), strawberry crinkle virus (SCV), and strawberry mild yellow edge virus (SMYEV) [8]. SMoV and SVBV are two of the most significant viruses affecting global strawberry production [8,9,10]. Both are latent viruses, making them difficult to detect in a timely manner due to their uneven distribution and low titer in host tissues. These characteristics limit the sensitivity and accuracy of conventional RT-PCR detection methods.

SMoV is a positive-sense single-stranded RNA (+ssRNA) virus that comprises two single-stranded RNA molecules [11]. The virus particles are spherical, with a diameter of 28–30 nm. RNA1 is 7036 nucleotides (nt) in length, and RNA2 is either 5619 nt or 6340 nt [1,12]. Numerous detection methods for SMoV have been developed. In the early stages, indicator plants were used for detection [8,13]. However, due to the limitations of the leaf-grafting method, this approach is now rarely used. Currently, RT-PCR is the primary method for SMoV detection [14,15]. Several researchers have developed multiplex RT-PCR systems capable of simultaneously detecting SMoV along with other strawberry viruses [16,17].

SVBV has spherical particles with a diameter of 40–50 nm and contains a single circular double-stranded DNA (dsDNA) molecule of approximately 7.8 kb, including seven open reading frames (ORFs) [18]. Molecular techniques are now primarily employed for SVBV detection. In 2003, Sui Chun et al. [19] used both specific primer-based PCR and the leaf-grafting method to detect SVBV and obtained consistent results, confirming the accuracy of PCR-based detection. In 2016, Miao Lixiang et al. [20] established a SYBR Green I-based quantitative PCR (qPCR) system for SVBV, which demonstrated greater accuracy and sensitivity compared to conventional PCR.

TaqMan-based RT-qPCR is a highly specific and sensitive method for pathogen detection. It uses target-specific primers and a fluorescent probe with a reporter at the 5′ end and a quencher at the 3′ end [21,22]. During amplification, Taq polymerase degrades the bound probe, releasing a fluorescent signal only when the target is present, ensuring high specificity and accuracy [23]. The method also supports precise quantification, low contamination risk, and multiplex detection using different dyes [24]. Such a multiplex detection strategy holds significant value in virus detection, pathogen identification, and transgenic component analysis and is especially suitable for scenarios requiring rapid and accurate screening of multiple viruses, including plant viruses, animal viruses, and human pathogens in high-throughput settings.

Currently, the detection of strawberry viruses primarily relies on RT-PCR technology. However, this method has limitations and cannot quantify viruses within plant tissues. This is particularly challenging for strawberry viruses, which are typically present at low concentrations and unevenly distributed within the host, making stable detection more difficult [15]. RT-PCR also has certain shortcomings in terms of sensitivity and detection range. In contrast, RT-qPCR offers higher sensitivity and detection efficiency and, importantly, enables absolute quantification of viral RNA. Numerous previous studies have demonstrated that RT-qPCR generally provides greater sensitivity and higher detection rates than RT-PCR. Therefore, in this study, we established singleplex and duplex TaqMan RT-qPCR detection systems for SMoV and SVBV.

## 2. Results

### 2.1. Amplification of SMoV and SVBV Templates

**Figure 1:**
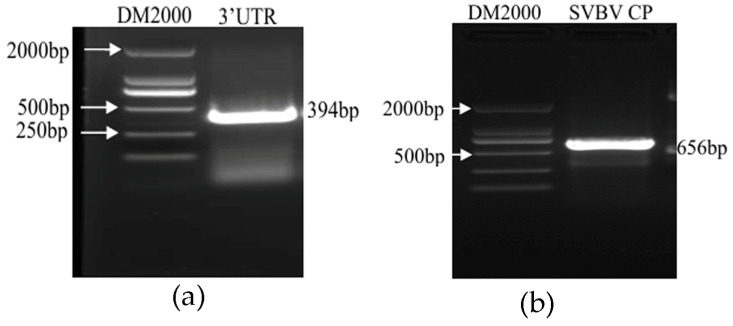
Preparation of templates: (**a**) Amplification of SMoV 3′UTR; (**b**) amplification of SVBV CP.

Preparation of the SMoV standard sample: A 394 bp target fragment was amplified via PCR using primers designed based on the conserved region of the SMoV 3′UTR, as shown in Figure 1a. The plasmid concentration was measured at 265.15 ng/μL, and its copy number was calculated to be 1.07 × 10^11^ copies/μL using the relevant formula.

Preparation of the SVBV standard sample: A 646 bp target fragment was amplified by PCR using primers designed from the SVBV CP region, as shown in Figure 1b. The plasmid concentration was 345.57 ng/μL, and the copy number was calculated to be 1.25 × 10^11^ copies/μL according to the formula.

### 2.2. Establishment of the Standard Curve for SMoV Singleplex TaqMan RT-qPCR

To determine the optimal concentrations of primers and probe for the SMoV TaqMan RT-qPCR assay, a recombinant plasmid containing the 3′ untranslated region (3′UTR) sequence of Strawberry Mottle Virus (SMoV) was used as the amplification template. A series of RT-qPCR reactions were conducted using different combinations of primer concentrations (ranging from 0.2 to 0.8 μmol/L) and probe concentrations (ranging from 0.1 to 0.5 μmol/L). The performance of each reaction was evaluated by comparing the resulting quantification cycle (Cq) values, as shown in Table 1. Although the differences in Cq values among the various combinations of primer and probe concentrations were relatively small, the reaction containing 0.8 μmol/L of each primer and 0.1 μmol/L of the TaqMan probe consistently produced the lowest Cq value, indicating the highest detection sensitivity. Based on these results, 0.8 μmol/L for the primers and 0.1 μmol/L for the probe were selected as the optimal conditions for subsequent SMoV RT-qPCR assays.

**Figure 2:**
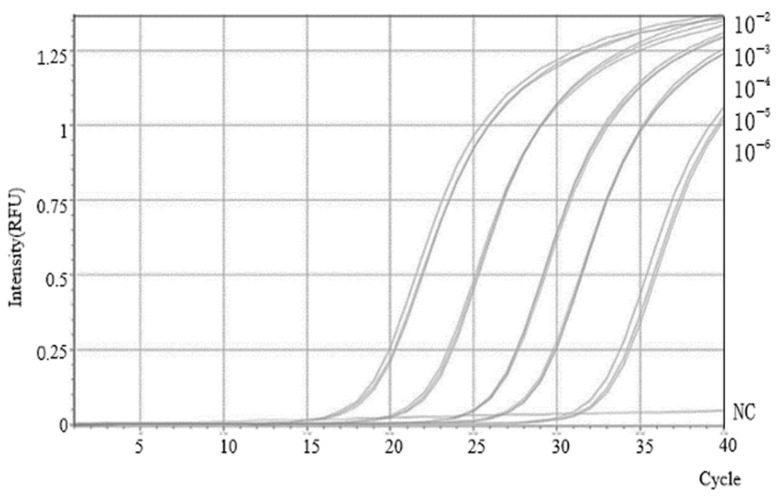
Establishment of the SMoV TaqMan RT-qPCR standard curve. RT-qPCR amplification curves for SMoV RNA quantification, with copy numbers ranging from 10^9^ to 10^5^ corresponding to dilutions from 10^−2^ to 10^−6^.

To determine the sensitivity of the SMoV RT-qPCR assay, a series of 10-fold serial dilutions of plasmid DNA were prepared, ranging from 10^9^ to 10^5^ copies/μL. RNase-free water was included as a negative control to rule out contamination or nonspecific amplification. Reliable amplification was still observed at the lowest concentration of 10^5^ copies/μL, corresponding to a Cq value of approximately 31, which was thus defined as the detection limit of the system. As shown in Figure 2, there was a strong linear relationship between the logarithm (base 10) of the template copy number and the corresponding Cq values. As shown in the Supplementary Figure S1, a standard curve for SMoV was established. Linear regression analysis yielded the equation: Cq = −3.43 log_10_ (q) + 11.12, with a correlation coefficient (R^2^) of 0.9926, indicating excellent linearity and reproducibility over a wide dynamic range of template concentrations.

### 2.3. Sensitivity Analysis of the SMoV Singleplex TaqMan RT-qPCR Assay

**Figure 3:**
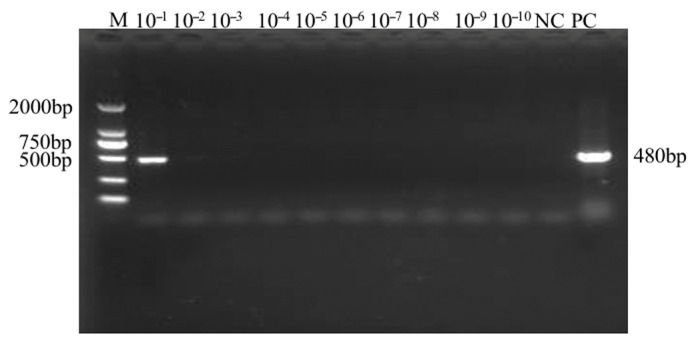
Sensitivity detection of SMoV by RT-PCR. NC represents the negative control, PC the positive control, and M indicates the 2000 bp DNA marker. Samples labeled 10^−1^ to 10^−10^ correspond to serial dilutions of the template. The expected amplicon size is 480 bp.

A gradient of ten standard plasmid templates with concentrations ranging from 10^−1^ to 10^−10^ (corresponding to 10^10^ to 10^1^ copies/μL) was used for the sensitivity comparison, with RNase-free H_2_O serving as the negative control. The minimum detectable copy number, using the conventional RT-PCR, was 10^10^ copies/μL from Figure 3. In contrast, the SMoV TaqMan RT-qPCR assay achieved a detection limit as low as 10^5^ copies/μL, demonstrating a sensitivity that is 10^5^ times higher than that of conventional RT-PCR. To evaluate repeatability, a standard template with a concentration of 10^9^ copies/μL was selected as the positive control. The assay was performed in triplicate across three independent runs, yielding a total of nine Cq values from Table 2. Intra-assay and inter-assay analyses revealed coefficients of variation (CVs) of less than 5%, indicating excellent repeatability and high reliability of the established SMoV TaqMan RT-qPCR method.

### 2.4. Establishment of the SVBV Singleplex TaqMan RT-qPCR Standard Curve

To determine the optimal concentrations of primers and probe for the SVBV-TaqMan RT-qPCR assay, recombinant plasmids containing the SVBV CP sequence were used as amplification templates. A series of RT-qPCR reactions were performed using different combinations of primer concentrations (ranging from 0.2 to 0.8 μmol/L) and probe concentrations (ranging from 0.1 to 0.5 μmol/L). The performance of each reaction was evaluated by comparing the resulting quantification cycle (Cq) values, as shown in Table 3. Although the differences in Cq values among the various primer and probe combinations were relatively small, the reaction containing 0.6 μmol/L of each primer and 0.1 μmol/L of the TaqMan probe consistently produced the lowest Cq value, indicating the highest detection sensitivity. Based on these results, 0.6 μmol/L primers and 0.1 μmol/L probe were selected as the optimal conditions for subsequent SVBV detection.

**Figure 4:**
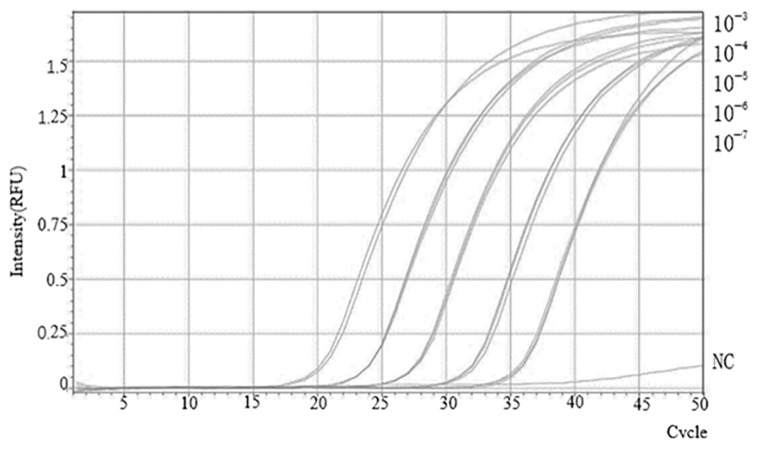
Establishment of the SVBV TaqMan RT-qPCR standard curve. RT-qPCR amplification curves for SVBV RNA quantification, with copy numbers ranging from 10^8^ to 10^4^ corresponding to dilutions from 10^−3^ to 10^−7^.

To determine the sensitivity of the SVBV RT-qPCR assay, the standard template was serially diluted over a range from 10^8^ to 10^4^ copies/μL and used as the positive control, while RNase-free water was included as a negative control to exclude contamination or nonspecific amplification. The Cq value for detecting the transcription level of SVBV was 34, which was defined as the detection limit of the system. As shown in Figure 4, there was a linear relationship between the Cq value and the logarithm of the template concentration. The standard curve of SVBV RT-qPCR is shown in Supplementary Figure S2.

### 2.5. Sensitivity Analysis of the SVBV Singleplex TaqMan RT-qPCR Assay

**Figure 5:**
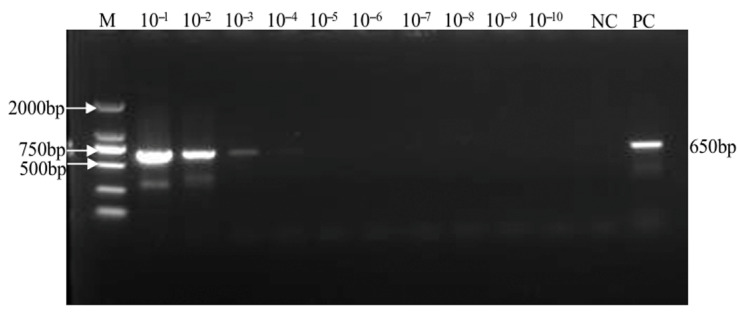
Sensitivity detection of SVBV via PCR. NC represents the negative control, PC the positive control, and M indicates the 2000 bp DNA marker. Samples labeled 10^−1^ to 10^−10^ correspond to serial dilutions of the template. The expected amplicon size is 650 bp.

A gradient of ten standard templates with concentrations ranging from 10^−1^ to 10^−10^ (corresponding to 10^10^ to 10^1^ copies/μL) was used for sensitivity comparison, with RNase-free H_2_O as the negative control. The minimum detectable copy number by conventional RT-PCR was 10^8^ copies/μL from Figure 5, while the detection limit of the SVBV TaqMan RT-qPCR assay was as low as 10^4^ copies/μL, indicating that the TaqMan RT-qPCR method is over 1000 times more sensitive than conventional RT-PCR. To assess repeatability, a standard template with a concentration of 10^8^ copies/μL was used as the positive control. The assay was performed in triplicate across three independent runs, yielding a total of nine Cq values from Table 4. Intra- and inter-assay analyses showed that the coefficients of variation were all below 5%, demonstrating excellent repeatability of the established SVBV TaqMan RT-qPCR method.

### 2.6. Establishment of the SMoV and SVBV Duplex TaqMan RT-qPCR Standard Curve

To optimize the duplex TaqMan qPCR system for the simultaneous detection of SMoV and SVBV, a fixed amount of SVBV and SMoV DNA was used, along with constant concentrations of SVBV primers (0.8 μmol/L) and probe (0.1 μmol/L). Meanwhile, the concentrations of SMoV primers and probes were optimized. A series of duplex RT-qPCR reactions were performed using different combinations of SMoV primer concentrations (ranging from 0.4 to 0.9 μmol/L) and probe concentrations (ranging from 0.1 to 0.3 μmol/L). As shown in Table 5, although the Cq value differences among various primer/probe combinations were relatively small, the combination of 0.9 μmol/L for each SMoV primer and 0.1 μmol/L for the probe consistently produced the lowest Cq value and the greatest increase in fluorescence signal intensity. Therefore, this combination was selected as the optimal primer and probe concentration for the duplex TaqMan RT-qPCR assay.

**Figure 6:**
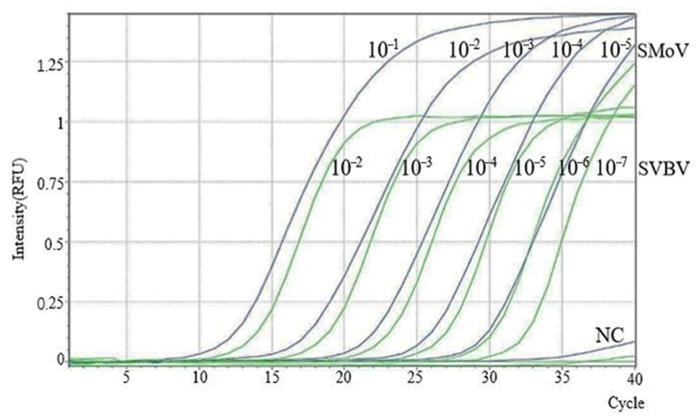
Amplification curves of RT-qPCR quantification for SMoV and SVBV RNA. The dilution series for SMoV (10^−1^ to 10^−5^) corresponds to 10^10^ to 10^6^ copies/μL, and for SVBV (10^−2^ to 10^−7^) corresponds to 10^9^ to 10^4^ copies/μL.

Two recombinant plasmids were serially diluted in 10-fold gradients to generate ten concentration levels: for SMoV, from 10^10^ to 10^6^ copies/μL; for SVBV, from 10^9^ to 10^4^ copies/μL. These two sets of plasmid standards were then used as templates to construct a duplex TaqMan RT-qPCR standard curve. RNase-free water served as the negative control template for both targets. Duplex TaqMan RT-qPCR reactions were performed using these templates. The results showed that when the threshold was reached, the number of amplification cycles between the concentrations of SMoV and SVBV showed an increasing trend in turn, the amplification curve reached a plateau period, and the results from Figure 6 were good. The detection limits of the system were determined to be 10^6^ copies/μL for SMoV (Cq = 28) and 10^4^ copies/μL for SVBV (Cq = 31). As shown in the Supplementary Figure S3, standard curves for SMoV and SVBV were established.

### 2.7. Sensitivity Analysis of the Duplex TaqMan RT-qPCR Assay for SMoV and SVBV

**Figure 7:**
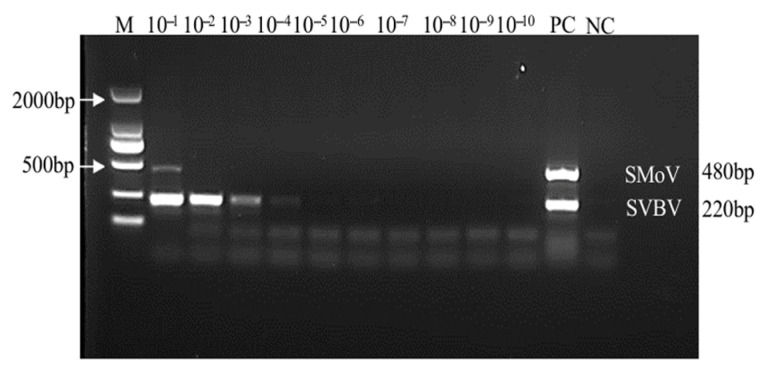
Duplex PCR sensitivity assay: NC represents the negative control, PC the positive control, and M indicates the 2000 bp marker. Samples labeled 10^−1^ to 10^−10^ correspond to serial dilutions. The expected amplicon size for SMoV is 480 bp, and it is 220 bp for SVBV.

Five standard templates with SMoV concentrations ranging from 10^10^ to 10^6^ copies/μL and six standard templates with SVBV concentrations ranging from 10^9^ to 10^4^ copies/μL were selected as positive templates, with RNase-free H_2_O as the negative control. The sensitivity comparison showed that the detection limit of the duplex RT-PCR method was 10^10^ copies/μL for SMoV and 10^8^ copies/μL for SVBV from Figure 7. In contrast, the minimum detectable concentration for the duplex TaqMan RT-qPCR assay was 10^6^ copies/μL for SMoV and 10^4^ copies/μL for SVBV. These results indicate that the duplex TaqMan RT-qPCR system developed in this study offers significantly higher sensitivity than duplex RT-PCR, with detection limits improved by at least 1000-fold for both viruses.

### 2.8. Comparison Between RT-PCR and Duplex TaqMan RT-qPCR Techniques on Field Samples

**Figure 8:**
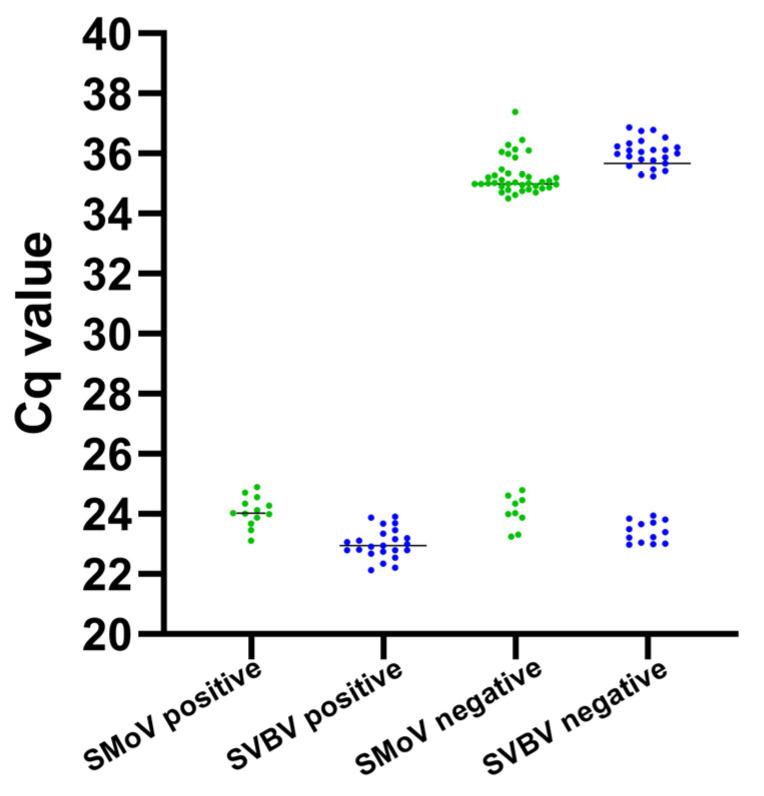
Comparison between RT-PCR and duplex TaqMan RT-qPCR techniques on field samples. The Cq values are plotted for RT-PCR-positive and RT-PCR-negative sample groups for each virus.

A total of 60 one-year-old ‘Benihoppe’ strawberry (*Fragaria × ananassa*) plants were collected from strawberry production fields in Changping District, Beijing. The sampled leaves exhibited a range of symptoms, including mottling, chlorosis, and curling. The results showed that, for SMoV detection, duplex TaqMan RT-qPCR identified 22 positive samples (detection rate: 36.7%), whereas conventional RT-PCR detected only 13 positive samples (detection rate: 21.7%). The detection rate of the new method was 1.7 times higher than that of the conventional method. For SVBV, the duplex TaqMan RT-qPCR detected 36 positive samples (detection rate: 60.0%), while the conventional RT-PCR detected 23 positive samples (detection rate: 38.3%), representing a 1.6-fold increase in the detection rate (Figure 8). This fully confirms that the duplex TaqMan RT-qPCR has a significantly higher detection efficacy in field virus monitoring.

## 3. Discussion

Many factors influence the performance of RT-qPCR assays, particularly due to the complex interplay and competitive interactions among primers, probes, and target nucleic acid fragments [25]. These factors can significantly impact the specificity, sensitivity, and overall efficiency of the amplification reaction. During primer and probe design, it is essential not only to ensure target specificity by avoiding cross-reactivity with non-target sequences but also to minimize the formation of undesirable secondary structures, such as hairpins or primer dimers that can lead to nonspecific amplification and signal interference. In this study, the singleplex TaqMan RT-qPCR assays for SMoV and SVBV could detect as low as 10^6^ copies/μL (genomic copies) and 10^4^ copies/μL (transcriptional level), respectively, demonstrating significantly higher sensitivity than conventional RT-PCR methods. As an RNA virus, the copy number of SMoV directly reflects the viral load, while SVBV is a DNA virus, and RT-qPCR detects its transcriptional products, which indicate the level of viral activity. The enhanced sensitivity observed for SVBV is primarily due to the detection of RNA transcripts rather than viral DNA.

In duplex RT-qPCR systems, where two sets of primers and probes are introduced simultaneously, these challenges are further amplified. The increased number of oligonucleotides raises the risk of unintended interactions and necessitates extensive optimization to determine the most effective concentration combinations that maintain sensitivity and efficiency for both targets [26]. In this study, the duplex TaqMan RT-qPCR system exhibited high sensitivity, detecting SMoV at nucleic acid levels as low as 10^5^ copies/μL and SVBV at transcriptional levels as low as 10^4^ copies/μL, which is significantly superior to the detection limits of conventional RT-PCR methods. The enhanced sensitivity is mainly attributed to the specific binding of the TaqMan probes, generating fluorescence signals, and the high precision of real-time monitoring technology. Although the duplex format slightly reduced the sensitivity for SMoV to some extent, the overall detection sensitivity still meets practical requirements and is sufficient for monitoring early virus infections.

Introducing an additional set of primers and probes (for SVBV) in the duplex format may partially suppress the amplification efficiency for SMoV, possibly due to competition for shared reaction components such as dNTPs, polymerase, or magnesium ions. This is a common observation in multiplex assays and underscores the need for careful balancing of reaction conditions. Despite the minor compromise in SMoV detection sensitivity, the duplex RT-qPCR system offers a major practical advantage: it enables the simultaneous detection of two economically important strawberry viruses (SMoV and SVBV) in a single reaction tube. This not only reduces reagent consumption and labor time but also improves throughput and consistency in routine diagnostics. Compared with conventional endpoint PCR techniques, the TaqMan-based RT-qPCR system developed in this study provides a number of significant improvements. The TaqMan-based RT-qPCR system developed in this study exhibits higher sensitivity, which is critical for monitoring infection severity, evaluating transmission potential, and assessing treatment or resistance strategies [27].

SMoV and SVBV are among the most prevalent and economically important viruses infecting cultivated strawberries [8]. While both viruses can remain latent in infected plants, they are often associated with significant yield losses, particularly under mixed-infection scenarios or in susceptible cultivars. SMoV infection has been reported to cause plant stunting, leaf chlorosis, and reduced fruit size and quality, while SVBV, though often asymptomatic when infecting alone, can lead to weakened plants and reduced runner production [28]. Therefore, early and accurate detection of these pathogens is critical for effective disease management, virus-free propagation, and certification of planting materials.

In conclusion, the duplex TaqMan RT-qPCR assay developed here is a rapid, sensitive, and reliable tool for the simultaneous detection of SMoV and SVBV and holds substantial potential for application in large-scale field surveillance, nursery screening, and quarantine inspection of strawberry planting materials. Future efforts may focus on further multiplexing to detect additional strawberry viruses or adapting the assay for portable, field-deployable platforms.

## 4. Materials and Methods

### 4.1. Plant Materials

The virus-infected strawberry samples used in this study were collected by our laboratory from Beijing, Shandong, Xinjiang, and Xuzhou. Approximately 0.5 g of fresh, tender leaf tissue from plants carrying SMoV and SVBV was selected. After rapid freezing in liquid nitrogen, the samples were stored at −80 °C.

### 4.2. Total RNA Extraction and Reverse Transcription from Plant Tissues

Total RNA was extracted from strawberry tissues using the OMEGA Total RNA Extraction Kit (OMEGA Bio-tek, Norcross, GA, USA), following the manufacturer’s protocol. The purity and concentration of the extracted RNA were determined using a NanoDrop micro-spectrophotometer (Thermo Fisher Scientific, Waltham, MA, USA), ensuring that the A260/A280 ratio was between 1.8 and 2.0. To eliminate potential genomic DNA contamination, the extracted total RNA was treated with DNase I. The DNase I-treated RNA was then used as a template for first-strand cDNA synthesis using M-MLV reverse transcriptase (Promega, Madison, WI, USA).

The reverse transcription reaction was performed in a total volume of 20 μL, consisting of the following components: 1 μL M-MLV reverse transcriptase (200 U/μL), 4 μL 5×M-MLV buffer, 1 μL Oligo (dT)_18_ (10 µmol/L), 1 μL RNase inhibitor (40 U/μL), 4 μL dNTP Mix (2.5 mM each), 1 μL random primers (10 μmol/L), 2 μL total RNA (500 ng), and 6 μL RNase-free H_2_O. The reaction mixture was gently mixed, briefly centrifuged, and incubated according to the enzyme manufacturer’s protocol (typically at 37 °C for 60 min, on ice for 5 min). The synthesized cDNA was either used immediately for subsequent RT-qPCR analysis or stored at −20 °C until further use.

### 4.3. Primer and Probe Design and Synthesis

The primer and probe sequences were derived from the SMoV and SVBV gene sequences available in NCBI. The primers for the target fragments of the SMoV 3′UTR and the CDS region of SVBV CP were designed using Primer 6.0 software (Primer, Vancouver, BC, Canada). Primers and probes for SMoV RT-qPCR and SVBV CP were designed using the IDT website (https://www.idtdna.com/page accessed on 7 September 2024). The 5′ end of the probe was labeled with FAM or VIC, and the 3′ end was labeled with TAMRA. The primers and probes for SMoV singleplex TaqMan RT-qPCR are listed in Supplementary Table S1, the primers and probes for SVBV singleplex TaqMan RT-qPCR are listed in Supplementary Table S2, and the primers and probes for SMoV and SVBV duplex TaqMan RT-qPCR are listed in Supplementary Table S3. The primers and probes were synthesized by Sangon Biotech Co., Ltd. (Shanghai, China).

### 4.4. Preparation of TaqMan RT-qPCR Standard Curve

The plasmid standard templates containing the target sequences were serially diluted using RNase-free H_2_O as the diluent. A 10-fold dilution series was prepared, covering ten concentration gradients ranging from 10^−1^ to 10^−10^, to generate a standard curve for quantitative analysis. Each dilution point was prepared in triplicate to ensure experimental reproducibility and statistical reliability. For the qPCR reactions, 2 μL of each diluted plasmid sample was used as the template. RNase-free H_2_O was included as a no-template control (NTC).

All reactions were performed using the Premix Ex Taq™ Probe qPCR Kit (Takara Bio Inc., Dalian, China), which contains a hot-start DNA polymerase and optimized buffer system for TaqMan probe-based detection. The reaction mixture typically consisted of 10 μL of 2× Premix Ex Taq, 1.6 μL forward and reverse primers, 0.2 μL TaqMan probe, 1.6 μL template DNA (or RNase-free H_2_O for controls), and RNase-free H_2_O to a final volume of 20 μL. Quantitative PCR was conducted using the MyGo Pro (IT-IS, Middlesbrough, UK) real-time PCR detection system under the following cycling conditions: initial denaturation at 94 °C for 30 s, followed by 50 cycles of denaturation at 94 °C for 5 s and annealing/extension at 60 °C for 30 s. Fluorescence data were collected at the end of each cycle. The resulting quantification cycle (Cq) values were used to generate standard curves by plotting the Cq values against the logarithm (base 10) of the initial template copy number. The linearity, amplification efficiency, and detection limit of the assay were assessed based on the standard curve parameters.

### 4.5. Singleplex TaqMan RT-qPCR

For singleplex TaqMan RT-qPCR, the 20 μL reaction included 1.6 μL of standard template for cDNA preparation via reverse transcription, 10 μL of Premix Ex Taq™ Probe qPCR Kit (Takara, Dalian, China), 1.6 μL of forward primer (10 µmol/L), 1.6 μL of reverse primer (10 µmol/L), 0.2 μL of probe (10 µmol/L), and 5 μL of double-distilled water. The amplification was performed using programmed reactions on a MyGo Pro (IT-IS, Middlesbrough, UK) real-time PCR system. Reaction Procedure: Pre-denaturation at 94 °C for 30 s, 1 cycle; RT-qPCR reaction: 94 °C for 5 s, 60 °C for 30 s, 40 cycles, with fluorescence signal acquisition. The final concentration of primers in the single detection was 0.8 µmol/L, and the final concentration of the probe was 0.1 µmol/L.

### 4.6. Duplex TaqMan RT-qPCR

For the duplex TaqMan RT-qPCR, the 20 μL reaction included 0.8 μL each of standard template for SMoV and SVBV cDNA (1.6 μL from the field samples) preparation via reverse transcription, 10 μL of Premix Ex Taq™ Probe qPCR Kit (Takara, Dalian, China), 0.9 μL of SMoV-qPCR forward primer (10 µmol/L), 0.9 μL of SMoV-qPCR reverse primer (10 µmol/L), 0.6 μL of SVBV-qPCR forward primer (10 µmol/L), 0.6 μL of SVBV-qPCR reverse primer (10 µmol/L), 0.1 μL of SVBV probe (10 µmol/L), 0.1 μL each of SMoV probe (10 µmol/L), and 5.2 μL of double-distilled water. The amplification was performed using programmed reactions on a MyGo Pro (IT-IS, Middlesbrough, UK) real-time PCR system. Reaction Procedure: Pre-denaturation at 94 °C for 30 s, 1 cycle; RT-qPCR reaction: 94 °C for 5 s, 60 °C for 30 s, 40 cycles, with fluorescence signal acquisition. The final concentration of SMoV primer was 0.45 µmol/L, SVBV primer was 0.3 µmol/L, and the final concentration of the two probes was 0.05 µmol/L.

### 4.7. PCR

Plasmid DNA obtained from Result 1 was used as the template for RT-PCR. The reactions were performed using Green Taq Mix (Vazyme, P131, Nanjing, China) in a total volume of 20 μL, containing 10 μL of Green Taq Mix, 1 μL of forward primer (10 µmol/L), 1 μL of reverse primer (10 µmol/L), 1 μL of template DNA, and 7 μL of nuclease-free water. The PCR amplification program was as follows: initial denaturation at 95 °C for 3 min; followed by 30 cycles of 95 °C for 15 s, 60 °C for 15 s, and 72 °C for 1 min; with a final extension at 72 °C for 5 min. PCR products were analyzed by agarose gel electrophoresis. A 1.5% agarose gel was prepared using Ultra GelRed nucleic acid stain (10,000×, Vazyme, Nanjing, China), and electrophoresis was performed in 1× TAE buffer. A DNA marker (DM2000, TsingKe Biotech, Beijing, China) was used as the molecular weight standard. Bands were visualized under UV light using a gel imaging system. The PCR reactions were performed using the Eppendorf Mastercycler^®^ nexus (Eppendorf, Hamburg, Germany).

### 4.8. Gene Cloning and Plasmid Extraction

Gene cloning was performed using the 2× KeyPo Master Mix (Dye Plus) (Vazyme, Nanjing, China) with high-fidelity DNA polymerase. The PCR program was as follows: 98 °C for 10 s, 60 °C for 5 s, and 72 °C for 5 s, for a total of 35 cycles. The gel-purified DNA products were transformed into Escherichia coli DH5α competent cells (Qingke Biotechnology, Beijing, China). Single colonies that tested positive by PCR screening were selected for overnight culture. Plasmid DNA was extracted using the TIANprep Mini Plasmid Kit (DP103; Tiangen Biotech, Beijing, China) according to the manufacturer’s instructions. The concentration and purity of the extracted plasmids were measured using a NanoDrop 2000 spectrophotometer (Thermo Fisher Scientific, Waltham, MA, USA). The plasmid samples were stored at −20 °C until further use.

## Figures and Tables

**Table 1:** The Cq value of single SMoV in the RT-qPCR assay using different primer and probe concentrations.

Primer Concentration (μmol/L)	Probe Concentration (μmol/L)				
	0.1	0.2	0.3	0.4	0.5
0.2	11.842	11.935	12.028	11.923	11.793
0.4	11.721	11.753	12.001	11.646	11.657
0.6	11.732	11.746	11.745	11.732	11.638
0.8	11.628	11.711	12.095	11.702	11.683

**Table 2:** Evaluation of the repeatability of the TaqMan RT-qPCR assay for the detection of SMoV.

Group	Cq Value			CV	
				Intra-Group	Inter-Group
1	17.659	18.047	17.937	1.11%	1.54%
2	18.934	17.564	18.332	3.75%	
3	17.892	18.457	18.921	2.79%	

**Table 3:** The Cq value of SVBV in the RT-qPCR assay using different primer and probe concentrations.

Primer Concentration (μmol/L)	Probe Concentration (μmol/L)				
	0.1	0.2	0.3	0.4	0.5
0.2	13.159	13.445	13.403	13.421	13.381
0.4	13.380	13.598	13.504	13.521	13.428
0.6	13.061	13.165	13.237	13.439	13.372
0.8	13.363	13.461	13.470	13.377	13.600

**Table 4:** Evaluation of the repeatability of the TaqMan RT-qPCR assay for the detection of SVBV.

Group	Cq Value			CV	
				Intra-Group	Inter-Group
1	19.347	19.669	18.584	2.90%	2.77%
2	17.653	18.418	18.614	2.79%	
3	18.349	17.893	19.012	3.06%	

**Table 5:** The Cq value of Duplex SMoV in the RT-qPCR assay using different primer and probe concentrations.

Primer Concentration (μmol/L)	Probe Concentration (μmol/L)				
	0.1	0.15	0.2	0.25	0.3
0.4	30.056	30.302	30.542	30.925	32.490
0.5	29.411	28.870	29.394	31.541	31.631
0.6	28.229	28.602	29.049	30.189	30.527
0.7	29.587	28.199	30.387	29.463	30.051
0.8	27.300	28.835	28.963	37.628	30.428
0.9	26.444	27.603	29.154	30.388	33.390

## Data Availability

The original contributions presented in this study are included in the article/Supplementary Material. Further inquiries can be directed to the corresponding author.

## Supplementary Materials

The following supporting information can be downloaded at https://www.mdpi.com/article/10.3390/plants14152330/s1. Figure S1: RT-qPCR standard curve for SMoV RNA quantification. Figure S2: RT-qPCR standard curve for SVBV RNA quantification. Figure S3: Duplex RT-qPCR standard curves for quantification of SMoV (a) and SVBV (b). Table S1. Primers and Probes Used for Establishing the SMoV Single TaqMan RT-qPCR Detection System. Table S2. Primers and Probes Used for Establishing the SVBV Single TaqMan RT-qPCR Detection System. Table S3. Primers and Probes Used for Establishing the SMoV and SVBV Dual TaqMan RT-qPCR Detection System.
