# Supplementary material for: Establishment of Singleplex and Duplex TaqMan RT-qPCR Detection Systems for Strawberry Mottle Virus (SMoV) and Strawberry Vein Banding Virus (SVBV)
Source: Plants (Basel). 2025 Jul 27;14(15):2330. doi: 10.3390/plants14152330 (PMC12349619; doi:10.3390/plants14152330)
Supplement: Supplementary file 1 [file plants-14-02330-s001.zip › plants-3681650-Supplementary Figure.pdf]

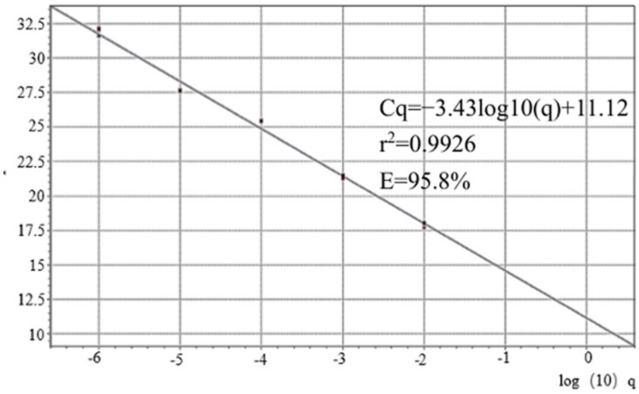


Supplementary Figure S1：RT-qPCR standard curve for SMoV RNA quantification.

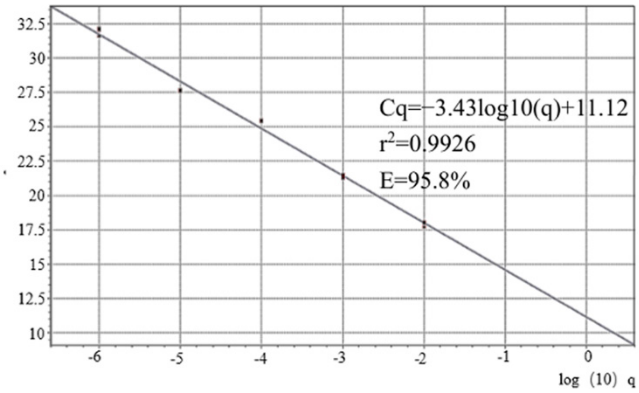


Supplementary Figure S2：RT-qPCR standard curve for SVBV RNA quantification.

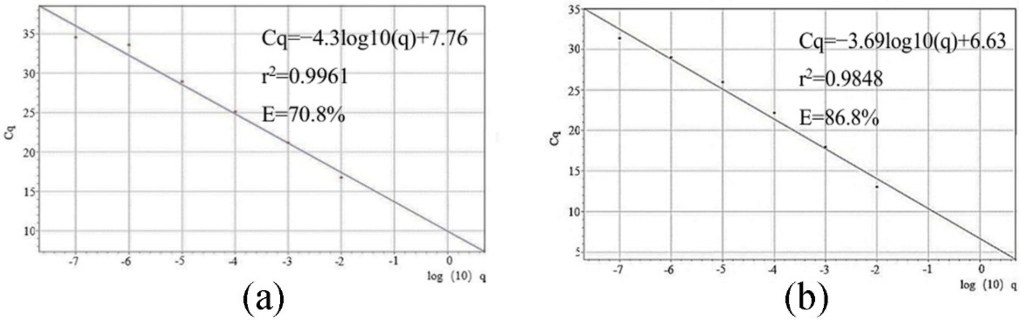


Supplementary Figure S3：Duplex RT-qPCR standard curves for quantification of SMoV (a) and SVBV (b).
