# Supplementary material for: Establishment of Singleplex and Duplex TaqMan RT-qPCR Detection Systems for Strawberry Mottle Virus (SMoV) and Strawberry Vein Banding Virus (SVBV)
Source: Plants (Basel). 2025 Jul 27;14(15):2330. doi: 10.3390/plants14152330 (PMC12349619; doi:10.3390/plants14152330)
Supplement: Supplementary file 1 [file plants-14-02330-s001.zip › plants-3681650-Supplementary Table.pdf]

Supplementary Table S1. Primers and Probes Used for Establishing the SMoV Single TaqMan RT-qPCR Detection System

Primer/probe	Sequence (5′-3′)	TM/℃	Note
SMoV UTR-F	GAACCGAATTTCACCAACCCT	55	*Sph* Ⅰ
SMoV UTR-R	CTACAGTTAGAATGCCGAAACT	54	*Pst* Ⅰ
SMoV RT-qPCR-F	AGCCGGGTTGTCAATTGTCTTT	58	
SMoV RT-qPCR-R	CTCCAGAGTGACCAAGCCTGA	54	
SMoV probe	CACCATGGGGAGGCTTGATGAGCCCA	63	5′FAM or VIC<br>3′TAMRA
SMoV RT-PCR-F	ATTTACATGTTGTAGTTTAGTG	50	
SMoV RT-PCR-R	AGTGGGTCACTACTCCACTA	52	

Supplementary Table S2. Primers and Probes Used for Establishing the SVBV Single TaqMan RT-qPCR Detection System

Primer/probe	Sequence (5′-3′)	TM/℃	Note
SVBV tranF	GAAAGACTCGAACAACTGTT	56℃	
SVBV tranR	GTAATTTGACTAACCTGTTTCTAG	54℃	
SVBV RT-qPCR-F	GACGAAGACACCTATCGTCCGAA	56℃	
SVBV RT-qPCR-R	CGGTTGCTTGCACAATCCAGAT	55℃	
SVBV probe	GCGACGAGGAACAAGCTCCTCTCAG CC	60.9℃	5′VIC<br>3′BHQ-1
SVBV RT-PCR-F	GATTAGCTACAGCAGCAGCA	55	
SVBV RT-PCR-R	GTACTTCCATCCCTACAAAG	54	

Supplementary Table S3. Primers and Probes Used for Establishing the SMoV and SVBV Dual TaqMan RT-qPCR Detection System

Primer/probe	Sequence (5′-3′)	TM/℃	Note
SMoV RT- qPCR-F	AGCCGGGTTGTCAATTGTCTTT	58	
SMoV RT- qPCR-R	CTCCAGAGTGACCAAGCCTGA	54	
SVBV RT- qPCR-R	GGATTGTGCAAGCAACCGAA	57	
SVBV RT- qPCR-R	GCTTGTTTGAAGGCATCAGTGT	58	
SMoV probe	CACCATGGGGAGGCTTGATGAGCCC A	62	5′FAM<br>3′TAMRA
SVBV probe	CAGTTGGCAATGCCAAACAATTGTT GG	61	5′VIC<br>3′BHQ-1
SMoV RT-PCR-F	ATTTACATGTTGTAGTTTAGTG	50	
SMoV RT-PCR-R	AGTGGGTCACTACTCCACTA	52	
SVBV RT-PCR-F	ATGAAGCTGCAAATGTCAAA	52	
SVBV RT-PCR-R	TTGTCCCATTCTTCAATGAG	52	
